# Role of NETosis in Central Nervous System Injury

**DOI:** 10.1155/2022/3235524

**Published:** 2022-01-04

**Authors:** Yituo Chen, Haojie Zhang, Xinli Hu, Wanta Cai, Wenfei Ni, Kailiang Zhou

**Affiliations:** ^1^Department of Orthopaedics, The Second Affiliated Hospital and Yuying Children's Hospital of Wenzhou Medical University, Wenzhou 325027, China; ^2^Zhejiang Provincial Key Laboratory of Orthopaedics, Wenzhou 325027, China; ^3^The Second Clinical Medical College of Wenzhou Medical University, Wenzhou 325027, China

## Abstract

Central nervous system (CNS) injury is divided into brain injury and spinal cord injury and remains the most common cause of morbidity and mortality worldwide. Previous reviews have defined numerous inflammatory cells involved in this process. In the human body, neutrophils comprise the largest numbers of myeloid leukocytes. Activated neutrophils release extracellular web-like DNA amended with antimicrobial proteins called neutrophil extracellular traps (NETs). The formation of NETs was demonstrated as a new method of cell death called NETosis. As the first line of defence against injury, neutrophils mediate a variety of adverse reactions in the early stage, and we consider that NETs may be the prominent mediators of CNS injury. Therefore, exploring the specific role of NETs in CNS injury may help us shed some light on early changes in the disease. Simultaneously, we discovered that there is a link between NETosis and other cell death pathways by browsing other research, which is helpful for us to establish crossroads between known cell death pathways. Currently, there is a large amount of research concerning NETosis in various diseases, but the role of NETosis in CNS injury remains unknown. Therefore, this review will introduce the role of NETosis in CNS injury, including traumatic brain injury, cerebral ischaemia, CNS infection, Alzheimer's disease, and spinal cord injury, by describing the mechanism of NETosis, the evidence of NETosis in CNS injury, and the link between NETosis and other cell death pathways. Furthermore, we also discuss some agents that inhibit NETosis as therapies to alleviate the severity of CNS injury. NETosis may be a potential target for the treatment of CNS injury, so exploring NETosis provides a feasible therapeutic option for CNS injury in the future.

## 1. Introduction

Neutrophils, which originate from hematopoietic stem cells, account for almost 50-70% of all white blood cells. Neutrophils are the primary line of defence of the innate immune system to fight against infection. These inflammatory cells are extensively involved in all kinds of inflammatory reactions. Once the host is infected by microorganisms, neutrophils immediately move from the bloodstream to the target of infection, serving as a “first line” of defence against foreign invaders. This process is called innate immunity. Neutrophils display antimicrobial functions through phagocytosis, degranulation, and NETs (neutrophil extracellular traps). NETs contain modified chromatin with histones and granular proteins, which kill microbes through oxidative or nonoxidative mechanisms and propagate inflammatory and immune responses [1]. The formation of NETs accompanied by cell death is called NETosis, which was first reported by Takei et al. [2]. NETosis is produced by immune complexes, microcrystals, antibodies, chemokines, cytokines, and physiological factors [3]. In recent studies, two different types of NETosis have been discovered: suicidal NETosis and vital NETosis [1]. Suicidal NETosis will cause cell death after the release of NETs, while cells remain alive in vital NETosis. These two different types of NETosis have their own functions and play an important role in immune defensive reactions.

CNS injury is one of the most serious organ injuries and has been very common worldwide in recent years [4]. According to clinical need, CNS injury comprises many types. CNS injury even causes paralysis in severe cases, seriously influencing individuals' physical and psychological health. Therefore, understanding with the mechanisms of neurotrauma and inventing effective pharmaceutical therapies are vital to improve patients' quality of life. Many cells are involved in the process of CNS injury, including meningeal cells, Schwann cells, endothelial cells, astrocytes, fibroblasts, pericytes, microglia, and other glia cells, which collectively attenuate damage or prompt repair [5]. CNS cells release DAMPs (damage-associated molecular patterns) after injury, which are sensed by macrophages or microglia. Admittedly, injury is a cause of inflammation activation. Damage of the CNS results in inflammatory responses from peripheral immune cells (such as T cells, monocytes, and neutrophils), macrophages, and resident microglia [6]. In addition, the migration of macrophages to injured areas and microglial activation are due to stimulation by chemokines, proinflammatory cytokines, or CNS environmental alterations [7]. Activation of macrophages and microglia has been proven to be related to CNS injury [8]. Neuroinflammation is a sign of spinal cord and brain injury. A large number of endogenous microglia and systemic macrophages migrate to injury sites to assist in clearing cellular debris after stroke, SCI, or TBI. In addition, CNS macrophages have the capability to dichotomously facilitate repairs while concurrently causing injury deterioration [8]. Neutrophils are the earliest inflammatory cells arriving at the injured region, and they possess a variety of toxic compounds. Studies show that CNS infiltration induced by neutrophils under different pathological conditions, such as brain ischaemia, trauma, neurodegeneration, infection, and autoimmunity, is a prominent phenomenon [9]. Previous studies have reported that classic cell death types, including autophagic cell death [10], apoptosis [11], necrosis [12], and pyroptosis [13], are involved in the process of CNS trauma. Macrophages are now widely researched in pyroptosis, while apoptosis occurs in neurons during invasion by viruses or other pathogens. In autophagy and necroptosis, we found neurons, astrocytes, and microglia implicated after CNS trauma [7]. Many cells mediated by various pathways of cell death have been identified in injury diseases, but the role of NETosis by neutrophils in CNS trauma remains unclear. We reasonably assume that NETosis probably regulates the mechanism of injury during inflammation. In this review, we summarize recent studies of NETosis in CNS trauma to provide a feasible future therapeutic method for CNS injury.

## 2. Mechanism of NETosis

As a specific type of cell death, NETosis is characterized by the release of web-like DNA structures decorated with histones and cytotoxic proteins from activated neutrophils [14]. This substance called NETs is triggered by microcrystals, cholesterol, antibodies, specific cytokines, and pharmacological stimuli containing calcium, potassium ionophores, and PMA [15]. The formation of NETs needs a variety of substances, such as NADPH oxidase, myeloperoxidase (MPO), c-Raf, MEK, ERK, and ROS (reactive oxygen species) [16]. NETosis is divided into suicidal NETosis and vital NETosis. In both, the nuclear membrane dissolves and decondensed chromatin exits the cell mixed with granular antimicrobial factors under the influence of all kinds of enzymes, which collectively form NETs [17]. After that, NETs trap almost all pathogens, even those that cannot be phagocytosed (such as viruses, yeasts, and protozoan parasites), killing them with a cocktail of bactericidal enzymes [3, 18]. Suicidal NETosis extrudes NETs accompanied by cell death; in vital NETosis, the cell is viable and retains its effector functions. Moreover, vital NETosis is induced by bacterial-specific molecular features identified by individual pattern recognition receptors (PRRs), whereas suicidal NETosis is typically attributed to PMA [19]. With regard to this newly described form of NETosis, we lack corresponding studies detailing its exact mechanism.

### 2.1. Suicidal NETosis

**Figure 1:**
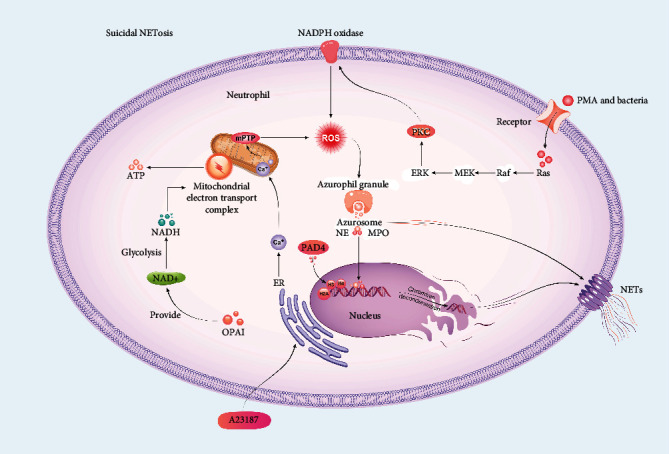
Mechanism of suicidal NETosis and induction of NETosis. PMA or bacteria combine with receptors and activate Ras and then activate PKC through the Raf-MEK-ERK pathway. PKC induces production of ROS by NADPH oxidase. ROS trigger rupture of azurephil granules and release azurosomes, which include NE and MPO. NE and MPO implicated in decondensation of chromatin. Finally, NETs are released through formation of GSDMD. A23187 induces an increase in Ca^2+^ concentration in mitochondria, which activate the opening of mPTP and produce mtROS. PAD4 moves into the nucleus and catalyses citrullination of histones (H3, H2A, and H4), through which it induces chromatin decondensation. OAP1 maintains the supply of NAD+, which later turns into NADH under the influence of glycolysis, and finally, NADH transfers electrons in the mitochondrial electron transport complex, which provides ATP for NET formation.

In 1996, Takei et al. found a new method of cell death, differing from necrosis and apoptosis, that is caused by phorbol 12-myristate 13-acetate (PMA) [2]. Subsequently, this method of cell death has been named suicidal NETosis [14]. This form of suicide includes nuclear swelling, chromatin decondensation, membrane perforation, and spilling of the nucleoplasm into the cytoplasm (Figure 1). In vivo, many kinds of pathogens trigger suicidal NETosis. First step is formation of NETs. Recent studies revealed the mechanism of NET formation. Fuchs et al. [20] speculated that ROS (including superoxide anions, dioxygen, and hydrogen peroxide) produced by NADPH oxidase may participate in NET formation and by using detailed cellular experiments in vitro. In his research, inhibition of NADPH oxidase by diphenylene iodonium (DPI) impaired the formation of NETs normally induced by PMA and live bacteria [20]. In addition, hydrogen peroxide was exogenously generated by glucose oxidase (GO) and used to stimulate neutrophils downstream of NADPH oxidase. The results indicated that the stimulation of hydrogen peroxide (membrane permeable) could produce NETs. In addition, studies have demonstrated that the Raf-MEK-ERK pathway activates protein kinase C (PKC), leading to membrane assembly with NADPH oxidase (Figure 1) [21, 22]. Another activation mechanism of NADPH oxidase in neutrophils relates to the ROS-dependent relationship between the p47phox subunit and the disulfide isomerase protein, which results in its migration onto the membrane to induce the release of NADPH oxidase [23]. In addition to ROS induced by NADPH oxidase, researchers also noticed an interesting phenomenon in which chronic granulomatous disease (CGD) patients, who lack NADPH oxidase to produce ROS, still demonstrate NET formation caused by A23187 but not PMA [15]. A23187 is a calcium ionophore that increases the intracellular Ca^2+^ concentration. Further studies indicated that Ca^2+^ overload induced by A23187 leads to increased mitochondrial ROS (mtROS) generation, while mtROS is not only cause NETosis by activating NADPH oxidase but also other pathways that do not involve NADPH oxidase, and mtROS is produced under the influence of the mitochondrial permeability transition pore (mPTP) (Figure 1) [15, 20, 24, 25]. These discoveries explain why CGD patients are able to also demonstrate NETosis. Generation of ROS leads to tubulin and actin glutathionylation regulated by glutaredoxin 1 (Grx1) [26]. Grx1 is a required enzyme during the process of microtubulin and actin deglutathionylation [26, 27]. Stojkov et al. and Amini et al. [26, 27] thought that an intact cytoskeleton is required for the formation of NETs. Lack of Grx1 leads to impaired cytoskeletal dynamics and causes defective degranulation. Additionally, active cytoskeletal rearrangements and glycolytic ATP production through an entire cytoskeleton are required elements in the formation of NETs. Successful NET formation requires energy from ATP released by glycolysis in the cytoplasm; since OPA1, as an inner mitochondrial membrane protein, maintains the supply of NAD+, NET formation also is indirectly regulated by OPA1 [26, 27]. The shortage of OPA1 leads to a decrease in mitochondrial electron transport complex I activity in neutrophils and consequently reduces ATP generation via glycolysis because of the lack of NAD+ [26, 27]. NET formation and the active assembly of the cytoskeletal network require OPA1-dependent ATP production (Figure 1) [26, 27]. Additionally, ROS participate in the dissociation of azurosomes, which are from Azurophil granules [28]. Azurosomes contain 8 categories of proteins. Three types of the proteins are serine proteases with high homology, namely, cathepsin G and azurocidin, neutrophilic elastase (NE), and myeloperoxidase (MPO) [28]. MPO and NE are associated with NETosis because they are implicated in the decondension of chromatin and the breakdown of cytoskeletal elements [28]. Under the influence of various mechanisms, NE in cytoplasmic granules migrates to the nucleus, incites nuclear envelope damage, and degrades chromatin by histone cleavage (Figure 1). Furthermore, myeloperoxidase (MPO) plays a role in decondensing nuclear DNA, but NET formation is independent of its enzymatic activity at this stage. It has been reported that the decondensation of chromatin is induced by MPO through enhancing the uncoiling of chromatin; however, the molecular mechanism is still unclear [29]. In addition, posttranslational modification may also contribute to the process of chromatin decondensation. Since posttranslational modifications occur in histone proteins (e.g., methylation, acetylation, and phosphorylation), various chromatin functions are altered, such as DNA damage repair, transcription, and the condensation/decondensation of chromatin [30, 31]. Through an HL-60 cell model, Wang et al. [32] indicated that peptidylarginine deiminase 4 (PAD4) in the cytoplasm migrates into the nucleus and catalyse histone citrullination, causing the decondensation of chromatin. PAD4, which is highly expressed in peripheral blood neutrophils, predominantly moves into the nucleus and is aimed at citrullinate histones H2A, H3, and H4. Inhibition of PAD4 Cl-amidine prevented chromatin from decondensing; neutrophils from PAD4 knockout mice and NETosis induced by Shigella flexneri or Ca^2+^ ionophores in HL-60 cells did not lead to the response of NETosis when induced by PMA [32–35]. It is found that PMA prompts the expression of PAD4 in cells [36]. In addition, transfection with a mimic of miR-155 and an antagomiR-155 increased and decreased PAD4 mRNA expression, respectively, in neutrophils, indicating that miR-155 is a potent regulator of PAD4 in neutrophils [36]. This finding reveals that miR-155 controls PAD4-dependent NET formation [36]. PAD4 also requires a high concentration of calcium for its activation [37]. Finally, nuclear DNA and antimicrobial compounds are released to the extracellular environment, accompanied by cell death via pores formed in the plasma membrane. Gasdermin D protein (GSDMD) contributes to the formation of pores on the plasma membrane (Figure 1). In contrast to pyroptosis in macrophages, typical NETosis hallmarks, involving histone citrullination, nuclear delobulation, DNA extrusion, the destruction of the plasma membrane, granules, and nucleus, which could be triggered after GSDMD activation. Previous studies demonstrated that GSDMD forms pores in the macrophage membrane during pyroptosis by activating caspase-1 and caspase-4/5, while it is activated and cleaved by NE in NETosis [38, 39]. This could also be accompanied by neutrophil lysis. Whether another mechanism causes neutrophil lysis after release of NETs remains unknown.

### 2.2. Vital NETosis

Suicidal NETosis needs cell death to play a role in antimicrobial function, but is there any other way to kill pathogens while keeping neutrophils alive? Some research groups have discovered a novel mechanism of NETosis without cell death. Pilsczek et al. found that neutrophils could distinctively respond to *Staphylococcus aureus* by a novel process of NET formation, during which neutrophil lysis and plasma membrane breakage were not required [40]. Morphologically, the multilocular neutrophil nucleus rapidly became round and compact. During the process, budding of vesicles and separation of the outer and inner nuclear membranes were observed, and the vesicles and separated membranes were filled with nuclear DNA. In addition to the mechanism whereby NETosis is caused by PMA, suicidal NETosis occurs much slower than vital NETosis, and this indicates that vital NETosis is a rapid mechanism [40]. Some studies have proven that platelet TLR4 recognizes TLR4 ligands in blood and binds to adherent neutrophils, contributing to the formation of NETs and the robustness of neutrophil activation [19, 41]. The NETs retained their integrity under flow conditions and ensnared bacteria within the vasculature. This phenomenon reveals that LPS may be an activator of rapid NETosis [41]. Whether a lack of nuclear DNA induces cell death is an important consideration. IFN-*γ*- or IL-5-primed eosinophils could rapidly respond to LPS by catapulting their DNA, which did not cause eosinophil apoptosis [42]. The authors explained that this phenomenon was primarily attributed to mitochondrial but not nuclear DNA extruded by eosinophils [42]. Therefore, we assume that neutrophils retain their viability after NETosis in the same manner as eosinophils. Interestingly, a group found that neutrophils treated with GM-CSF could then respond to C5a or LPS by emitting mitochondrial DNA due to oxidant mediation [1]. In this manner, a neutrophil could emit NETs, while its nucleus and other antimicrobial functions were retained. It is paradoxical to the outcome that vital NETosis does not need oxidants, and simultaneous centrifugation of neutrophils forms nuclear cytoplasts, which is cryopreserved and maintain their ability to inactivate phagocytosed bacteria (e.g., *S. aureus*) and exhibit chemotaxis [43, 44]. Considering that erythrocytes live for more than 120 days without nuclei, we believe neutrophils also retain their function even if their nuclei are unavailable. Therefore, relevant studies are needed to describe this mechanism.

### 2.3. How NETs Play a Role in Immune Defence

NETs comprise numerous antimicrobial compounds, such as MPO and NE, which kill pathogens directly. As the most abundant proteins of NETs, histones possess a strong ability to kill microorganisms [45]. At the same time, as a component of NETs, DNA is also able to inhibit microorganisms. Generally, neutrophils are deemed to possess antimicrobial ability through phagocytosis, but recent studies show that neutrophils whose phagocytosis function was inhibited via treatment with cytochalasin D retained bactericidal activities and failed to function when treated with DNase or antihistone antibodies against H2A [3]. However, the considerable mechanism by which DNA and histones kill microbes is still unclear. Furthermore, physically attaching to pathogen cells and then trapping them are proposed as a mechanism for antimicrobial effects. The initial description of NETs was initially proposed following observations by electron microscopy and immunofluorescence [19]. Extracellular nucleic acids after NETosis stimulation have the ability to bind to exogenous *Salmonella typhimurium*, *S. aureus*, and *Shigella flexneri* [19]. Furthermore, in a model of rabbit shigellosis, bacteria were found to adhere to the NET structure [19]. In total, multiple bacteria have been shown to bind directly to extracellular DNA in vitro, including *Streptococcus pneumoniae*, *S aureus*, and *Escherichia coli* [19]. Therefore, we assume that the primary function of NETs is to trap bacteria, through DNA combinations, but it is not ruled out whether there are other methods to trap bacteria. In addition, components in NETs have also been proven to potentially kill bacteria. For example, histone H2B is able to penetrate through the E. coli membrane, histones H3 and H4 destroy the bacterial cell wall, NET-associated proteases (NE or PR3) inactivate and kill pathogens by cleaving their virulence factors, and LL37, a unique member of human cathelicidin antimicrobial peptides composed of 37 amino acids, disintegrates pathogen cell membranes, challenging pathogen viability; MPO is an effective antimicrobial material [46, 47]. Pretexts describe TLR4 function in NET formation. The activation of platelet TLR4 leads to a specific platelet response—specifically, attaching to adherent neutrophils in the sinusoids, resulting the formation of NETs, and enhancing the robustness of neutrophil activation [41, 48]. As a haemostatic regulator, platelet functions is only activated by very serious systemic infections that stimulate neutrophils to emit chromatin and proteolytic materials, thereby enhancing the innate immune system's ability to trap and kill microbial cells in circulation [41, 48]. Although LPS causes unconventional platelet activation, and the prevailing view is that during sepsis, the LPS emitted by bacteria activates neutrophils inappropriately and lock them in liver sinusoids and lung capillaries where the proteolysis improperly damages the tissues [41, 48]. It has also been reported that platelets are less sensitive to LPS than neutrophils, and NETs may damage the vascular endothelium [41, 48]. Therefore, NETs are vital to the immune defence response. However, when NETs are out of control, they may cause autoimmune diseases, such as systemic lupus erythematosus (SLE) [49–51]. DNase1 is important for the physiological degradation of NETs [49–51]. The degradation of PMA–NET induced by macrophages occurs in lysosomes [49–51]. If degradation of NETs is incomplete, residual NETs mediate inflammasome and complement activation; for example, lower concentrations of complement factors C3 and C4 were found in SLE patients, indicating consumption of complement and SLE-related NET complement activation, triggering the accumulation of C1q on NETs, which further inhibits DNase1 [52, 53]. Knowing the detailed defence mechanism of NETs in the immune system should be the focus of subsequent studies.

## 3. Linkage between NETosis and Other Cell Death Pathways

Programmed cell death mainly includes pyroptosis, necroptosis, apoptosis, and NETosis. There are many differences among these cell death pathways. In inflammatory cells, pyroptosis mainly occurs in specialized immune cells (such as macrophages, monocytes, and dendritic cells) and nonimmune cell types (such as intestinal epithelial cells, human trophoblasts, and hepatocyte cells), while NETosis takes place in the PMN [19, 54]. Morphologically, it was found that in NETosis, the cell membrane remains intact until the release of NETs, the cytoplasm remains unchanged or swollen, and the nucleus decondenses and mixes with cytoplasmic contents [55]. In apoptosis, the membrane blebs, but cell integrity is maintained, the cytoplasm becomes shrunk, and the nucleus condenses to form apoptotic bodies [55]. In necroptosis, the membrane loses its integrity, the cytoplasm becomes swollen, and the nucleus remains intact [55]. We explored whether there is a relationship between NETosis and these three cell death pathways. Neutrophils are involved in inflammation, so the formation of NETs is related to inflammatory reactions. Previous study indicated that inflammatory factors are implicated in the process of programmed cell death [56], so we predict that NETosis may trigger other death pathways by releasing NETs. Pyroptosis is an inflammatory form of cell death triggered by certain inflammasomes, leading to the cleavage of gasdermin D (GSDMD) and activation of inactive cytokines such as IL-18 and IL-1*β* [57]. DAMPs in pathogens are recognized by nucleotide oligomerization domain- (NOD-) like receptors (NLRs), which recruit caspase-1 to cleave pro-IL-18 and prointerleukin-1*β* (IL-1*β*) into their mature patterns and rupture GSDMD (encoded by GSDMD) to prompt pyroptosis and pore opening [56]. NE in NETs released during NETosis cleaves and activates GSDMD and induces neutrophil lysis [38, 39]. GSDMD could be a crosslink between pyroptosis and NETosis. The activation of the noncanonical inflammasome occurs during the recognition of LPS by caspase-4, stimulating its activation, caspase-1 activation, and GSDMD pore formation via the NLRP3 inflammasome. Noncanonical inflammasome stimulation through the transportation of LPS to the neutrophil cytoplasm activates caspase-4/-11 and causes the release of NETs in a GSDMD-dependent manner [58]. Related studies showed that NET-primed macrophages improved the IL-1*β*/Th17 response, causing atherogenesis [59]. IL-1*β* plays an important role in the canonical pyroptosis pathway, and NETosis may trigger pyroptosis by activating caspase-4 and IL-1*β*. In addition, a study has documented that apoptotic caspase-8 triggers the formation of GSDMD pores [60]. If NETs are able to activate caspase-8, NETs may trigger apoptosis [56]. In contrast to apoptosis, inhibition of caspase-8 induces necroptosis through recruiting and phosphorylating RIPK3 by RHIM-RHIM under the influence of continued RIPK1 kinase activity [56, 61, 62]. If NETs inhibit caspase-8 activity, NETosis may also cause necroptosis. Overall, GSDMD and caspase-8 may be central points in NETosis, pyroptosis, necroptosis, and apoptosis. Further studies are needed to prove this hypothesis.

## 4. Evidence of NETosis in CNS Injury

### 4.1. NETs in Traumatic Brain Injury (TBI)

TBI is often attributed to the application of mechanical force to the head. TBI has two stages. One is primary injuries, which occur at the time of impact. Another is secondary injuries developing under the circumstance of supervised medical care [63]. TBI is often considered to be involved in various chronic degenerative processes. After external violence, intracranial haemorrhage will result in increased neurological deterioration, cerebral hypoperfusion, and altered intracranial pressure (ICP) because the volume of the skull is limited. In addition to the influence of external factors and local compression by haematoma, inflammatory cells are also involved in the traumatic process. Therefore, as a diverse group of sterile injuries, TBI is generally induced by primary and secondary mechanisms, and it leads to neurologic dysfunction, inflammation, and cell death in patients of all demographics [64]. Studies have shown that after TBI, the CNS rapidly recruits neutrophils which enter through the choroid plexus and the vessels of meninges [65, 66]. As such, we confirm that neutrophils are implicated in the process of TBI, but whether NETs are required for the injury process remains to be further researched. Early signs of TBI pathogenesis are tissue destruction, hypoxia, and edema, through which the earliest soluble mediators, ROS, are produced [65]. As described in the pretext, ROS are critical mediators participating in the formation of NETs, so there are enough reasons to assume this hypothesis. Vaibhav et al. [67] discovered NETs in hypoperfused brain tissue through a mouse controlled cortical impact (CCI) model [67]. After that, to further explore the mechanism of NETs in TBI, researchers used C3H/HeJ mice lacking functional TLR4 compared to wild-type C3H/OuJ mice [67]. The results showed that C3H/HeJ mice displayed less NET formation and edema development after TBI. After TBI, the isolated C3H/OuJ neutrophils were transferred to either C3H/HeJ (WT>mutant (MUT)) mice or C3H/OuJ (wild type (WT)>WT) mice, which caused increases in NETs, indicating that TLR4 activation improves the generation of NETs [67]. Additionally, after TBI, TLR4 activation increased histone hypercitrullination and NET formation via a PAD4-dependent mechanism in isolated human neutrophils, and circulating levels of Cit-H3, a product of PAD4 activity, was noted [67]. Administration of Cl-amidine, an inhibiting agent of PAD4, dose-dependently reduced CNS neutrophil infiltration, attenuated NET production after TBI, reduced cerebral edema, improved CSF, and ameliorated neurological deficits after TBI. In addition, paroxysmal sympathetic hyperactivity (PSH) plays an important role in the high morbidity and mortality of TBI [67]. A research group used enzyme-linked immunosorbent assay with a rat diffuse axonal injury (DAI) model to evaluate the concentrations of plasma normetanephrine and metanephrine [68]. These values are indices of sympathetic system variation after TBI [68]. Study shows that after TBI, the levels of normetanephrine and metanephrine initially increased at 9 h and reached their highest values at 72 h [68]. After injury, in the paraventricular nucleus (PVN), a continuous increase in the concentration of NETs was observed at 24 and 72 h [68–71]. A positive connection between blood catecholamine and PVN or NET levels was found [68–71]. The flow cytometry assay of peripheral blood cells demonstrated that compared with the control group, there was a much higher level of NETs in the injury group [68–71]. The immunofluorescence results proved that NETs appeared in the PVN after TBI, the positive immunoprecipitation results indicated that LL37 was correlated with P2 × 7 [68–71]. As a portion of NETs, LL37 is an agonist of the P2 × 7 receptor, which is a trimer ATP-gated cationic channel and highly expressed on the cell membrane of microglia and monocytes [68–71]. LL37 could enhance IL-1*β* secretion in monocytes through the P2 × 7 receptor [68–71]. IL-1*β*, an important inflammatory mediator, takes part in sympathetic excitation by mediating neuronal activity and inhibitory/excitatory neurotransmitter levels [68–71]. The expression levels of IL-1*β* could be inhibited by PAD4 and Hippo/MST1 pathway inhibitors; furthermore, PAD4 inhibitors could also prevent MST1 expression [68–71]. This evidence reveals that IL-1*β* secretion is regulated by LL37 in NETs through the Hippo/MST1 pathway, under the influence of PSH, and microglial activation after TBI to eventually cause sympathetic hyperactivity. Moreover, since proteolytic proteins (e.g., metalloproteinases (MMPs) and cathepsin G and NE) are released, intravascular NETs directly lead to toxic effects on the endothelium [9]. MMPs and NE are related to the retraction of endothelial cells and the disruption of junctional complexes [9]. The permeability of endothelial cells was improved by NE itself, while the death of endothelial cells was caused by MPO and histones [9]. In this case, the production of NETs may damage cerebral vascular endothelial cells and brain cells, going a step further to exacerbate the destruction of the brain by hypoxia after TBI.

### 4.2. NETs in Cerebral Ischaemia

There are many reasons for cerebral ischaemia, including cerebral atherosclerotic plaque formation, cerebral artery thromboembolism, and cerebral artery stenosis. Chronic inflammation induced by neutrophils is considered to be a potential cause of atherosclerosis. A previous perspective is that the underlying pathophysiology termed atherosclerosis is a lipid-driven inflammatory disease of arteries developing at predilection sites with disturbed flow, in which endothelial activation contributes to intimal retention of lipoproteins [72]. Oxidized low-density lipoprotein and modified lipoproteins augment the damage to endothelial cells and cause the recruitment of leukocytes once they fail to pass through cell death, retain inflammation, and eliminate lipoproteins [72]. The growing lesions caused by chronic inflammation will induce vessel occlusion and subsequent arterial or ischaemic thrombosis [72]. Finding the linkage between NETs and atherosclerosis induced by chronic inflammation will further explain the pathophysiological mechanism. In research studies, NETs activated plasmacytoid dendritic cells in the vessel wall, leading to a strong response to type I interferon (IFN I), subsequently causing atherogenesis. After treatment with chloramidine to inhibit PAD4, thrombosis of the carotid artery and the size of atherosclerotic lesions were reduced in an atherosclerotic mouse model [73, 74]. In addition, NETs have a direct effect on inducing endothelial dysfunction (as a starting point of atherosclerosis) via endothelial cell activation and damage [75–77]. Some authors think that NET-mediated priming of macrophages induces PR3-mediated cytokine maturation or a strong IL-1*β*/Th17 response driving atherogenesis [59, 78]. Regardless, evidence reveals the potential correlation between NETs and cerebral atherosclerosis. Fuchs et al. have proved the relationship between NETs and thrombosis [79]. Extracellular DNA secreted by NETs served as a net for red blood cells and platelets, and high levels of histones and DNA render NETs highly procoagulant by activating and accumulating platelets [79]. Activated platelets adhere to procoagulant molecules, such as fibrinogen, von Willebrand factor (VWF), and fibronectin, which are immobilized on NETs [79]. Meanwhile, components in NETs also trigger procoagulant molecules or block inhibitors of the tissue factor pathway to accelerate the process of coagulation [80]. Moreover, it is well known that extracellular DNA induces coagulation and activates innate immune responses after infection [81]. Therefore, these studies showed that a variety of NET components present in the plasma of stroke patients are possibly required for NET prothrombotic effects and are assayed to assess disease severity. New studies are needed to further explore the relationship between thrombosis and NETosis in cerebral ischaemia. In addition, experiments by Kim and colleagues suggested that high-mobility group box-1 (HMGB1), a prototypic DAMP, is involved in NET-mediated neuronal damage in the ischaemic brain [82]. HMGB1 is a nonhistone chromosomal protein localized in the nucleus under normal physiological conditions that is considered to activate platelets and induce NETosis [82]. HMGB1 is not only emitted from NETosed neutrophils but also activated platelets [83–85]. The myocardial repair process is interfered with, by activated platelet-derived HMGB1 via decreasing the accumulation of mesenchymal stem cells through downregulating the hepatocyte growth factor receptor MET in a TLR4-dependent process [83–85]. Furthermore, NET formation and autophagy is enhanced by activated platelet-derived HMGB1 in a RAGE-dependent manner [83–85]. Therefore, NETosed neutrophil- or activated platelet-derived extracellular HMGB1 initiates thrombosis and further promotes NETosis, which mediates the aggravation cascade resulting in cerebral ischaemia and various other diseases associated with immunothrombosis [83–85]. In addition, NETosis caused by HMGB1 exacerbates ischaemic brain damage in the MACO model [82]. The results show that HMGB1 induces NETosis via TLR4 and CXCR4, which is consistent with the results described in the pretext that TLR4 is able to trigger the release of NETs [82]. When HMGB1 was emitted from neutrophils and aggregated in culture media and cell lysates, NETosis was triggered by circulating neutrophils treated with NCM, and those blood neutrophils treated with NCM were cocultivated with primary cortical neurons, increasing the levels of PI-positive neurons, suggesting that HMGB1 released from neutrophils after NETosis leads to NETosis-induced neuronal death and the presence of a reciprocal aggravating cycle between NETosis and neuronal cell death [82]. Some researchers also proved that the accumulation of ATP during cerebral ischaemia could lead to NETosis [86]. A prototypic P2X7R agonist (BzATP) or ATP dramatically induced CitH3 and PAD4 in a P2X7R-dependent manner, and NADPH oxidase-dependent ROS production, PKC*α* activation, and intracellular Ca^2+^ influx are critical in ATP-P2X7R-mediated NETosis [86]. Within the process of NETosis, the most effective component is NETs, so we assume damage in cerebral ischaemia is mainly ascribed to NETs. Many studies have shown an increase in NETs after cerebral ischaemia [87]. Kang et al. [87] through a middle cerebral artery occlusion (MACO) model indicated that stroke resulted in the accumulation of neutrophils in the brain, creating toxic signals (such as NETs), which subsequently improved the production of STING-dependent type I IFN-*β* activation. Remarkably, they also found that the increased generation and impaired clearance of NETs were detrimental to vascular repair and revascularization after stroke [87]. Additionally, active components in NETs destroy the viability of neurons. In vitro, coculturing transmigrated neutrophils with neurons for 3 h significantly reduced the viability of neurons [88]. However, DNase treatment of conditioned medium from transmigrated granulocytes did not evidently diminish the viability of neurons; therefore, the decrease in the viability of neurons was not ascribed to extracellular DNA [88]. Moreover, inhibiting neutrophil-derived extracellular proteases (such as neutrophilic proteases) related to NETs remarkably reduced neutrophil-mediated neurotoxicity [88]. Interestingly, as shown in experiments, the key neutrophilic proteases, cathepsin-G, NE, proteinase-3, and MMP-9, seem to attack neurons when a mixture of their inhibitors, but not any single specific inhibitor, nearly completely reversed the neutrophil-dependent neurotoxic effect [9, 88]. Altogether, these authors identified a novel neuroinflammatory mechanism: the development of rapid neurotoxicity of neutrophils initiated by IL-1-induced cerebrovascular transmigration [9, 88]. Consistently, Allen et al. [9, 88] proved that rapidly developed (30 min) neutrophil-dependent neurotoxicity is mediated by neutrophil-derived proteases released upon degranulation or associated with NETs. This result indicates that NETs may have toxic effects on neurons, which may be the reason for defects and nerve damage after stroke.

### 4.3. NETs in CNS Infection

CNS infection is uncommon in the presence of BBB (blood-brain barrier), but individuals may suffer from CNS infection, such as meningitis or encephalitis, under circumstances of low immunity. Local failure of the immune response mechanism leads to these devastating conditions, which consequently result in irreversible brain damage. The critical role of NETs in antimicrobial activity is clear [3], so we assume that NETs may participate in CNS infection. As described previously, NETs mainly kill bacteria through phagocytosis and oxidative stress as bactericidal mechanisms in CNS infection. In the clinic, neutrophils are observed crossing the BBB in bacterial meningitis upon the examination of CSF by lumbar puncture [89]. As described in the pretext, NETs capture both gram-positive and gram-negative microbes, and active components in NETs not only kill bacteria but also inhibit the spread of infection and retain homeostasis at the point of entry, even in unexpected body compartments [19]. By engulfing and immobilizing viruses, NETs also prevent virus entry into cells and spreading [90]. There are many antimicrobial compounds. Mohanty et al. [91] discovered that DNase1 significantly cleared bacteria in affected organs (lungs, brain, and spleen) and decreased bacterial viability in the presence of neutrophils in vitro. The eradication of bacteria from the brains of DNase-treated rats correlated with a decrease in IL-1*β* levels [91]. These findings indicated that DNA in NETs has antimicrobial activity. The antimicrobial activity effect was also annulled by inhibitors of phagocytosis, NADPH oxidase, and MPO [9, 91]. In addition, much evidence has revealed that NETs occur in CNS infection. From statistical data, increases in NETs were detected in children with enteroviral meningitis (EVM), tick-borne encephalitis (TBE), and Lyme neuroborreliosis (LNB), and the highest median levels of polynuclear cells were present in TBE [92]. Since monocytes play a primary role in viral encephalitis and are also able to release analogues of NETs, the authors determined the proportions of mononuclear and polynuclear cells in NET-positive samples [92]. The results confirmed that polynuclear cells are specifically related to NET levels rather than pleocytosis. In addition, the activity of NE and the levels of NETs displayed a high degree of correlation [92]. Therefore, they concluded that the NET structures mainly originated from neutrophils [92]. When the researchers tested chemokines and cytokines in CSF sample, the result showed that the levels of CXCL1, CXCL6, CXCL8, CXCL10, MMP-9, IL-6, and TNF were raised in the sample. Therefore, the recruitment of neutrophils into the CNS is a prerequisite in infections resulting from enterovirus and *B. burgdorferi*. The combination of CXC-receptor 2 on neutrophils and chemokines CXCL8, CXCL6, and CXCL1 could result in autoactivation and enable neutrophils to enter the CNS during infections [92]. The highest median levels of NETs/neutrophil-associated cytokines and chemokines were also determined in the CSF samples of children with EVM [92]. However, Mohanty et al. [91] noted that NETs hinder the clearance of bacteria in pneumococcal meningitis, and the application of DNase1 reduces pneumococcal viability in the brain and enhances the clearance of bacteria in a meningitis mouse model. Further data showed that suppression of NADPH, MPO, and phagocytosis caused the death of bacteria due to DNase treatment. Therefore, the authors assume that when encountering neutrophils, bacteria lead to NET generation in the brain; however, not all bacteria are associated with NETs [91]. Therefore, bound/unbound bacteria enhance their viable loading and induce leakage of the BBB. Ultimately, the bacteria go through the defective BBB and spread into other organs. Treatment with DNase degrades DNA in NETs, resulting in the release of bacteria and allows other defence mechanisms of neutrophils to control and promote the process of microbial clearance [91]. Elucidation of the relevant mechanisms still needs further research to provide appropriate future therapeutic targets.

### 4.4. NETs in Alzheimer's Disease (AD)

AD, which tends to occur in the elderly, is a neurodegenerative disorder characterized by the progressive degeneration of cognitive functions. Previous research indicated that the molecular pathogenesis of the disease is mainly ascribed to the loss of synapses and neurons, plaques composed of amyloid beta (Abeta), and tangles composed of hyperphosphorylated tau [93]. Chronic neuroinflammation is a typical feature of AD pathogenesis [94]. Since inflammation occurs in AD, we speculate that leukocytes may be implicated in the pathological process. Compelling evidence indicates that neutrophils are able to pass through the BBB and have been discovered inside the parenchyma and brain vessels of AD subjects. Moreover, recent data from two transgenic animal models of AD indicate that neutrophils bind to blood vessels and intrude into the brain parenchyma, causing neuropathological alterations and cognitive deficits [95]. Further studies have proven the production of NETs in an AD mouse model and in individuals with AD [94, 96]. A*β* is broadly considered a marker of Alzheimer's disease. A*β* promotes the generation of ROS by activating NADPH oxidase in both human and mouse neutrophils in vitro, and several reports have demonstrated that ROS production is a necessary step in the formation of NETs [9]. In addition to A*β*, some studies have demonstrated that NET formation in human neutrophils in vitro is also driven by the fibrillary form of amyloids from other sources, such as *α*-synuclein, Sup35, and transthyretin [9, 95]. In the same study, the presence of NETs was observed near amyloid deposits in patients with systemic amyloidosis, and NET-associated elastase was able to degrade amyloid fibrils into short toxic oligomeric species, suggesting that amyloid fibrils act as a reservoir of toxic peptides that may promote amyloid disease pathogenesis [95]. Studies have revealed that A*β*1–42 triggers the states of high affinity and intermediate affinity of LFA-1 (lymphocyte function-associated antigen 1) integrin, which modulates intraparenchymal motility and the intravascular adhesion of neutrophils [94, 96]. Then, A*β* improves the interaction between its endothelial ligands and LFA-1 [94, 96]. Although neutrophils were eliminated by injecting an anti-Ly6G antibody (clone 1A8) into the peripheral circulation of 6-month-old 3xTg-AD mice, the authors found that in comparison with the control group, the levels of cognitive function of mice in other groups were improved by treatment with anti-Ly6G [94, 96]. Cognitive deficits and pathology were induced due to inhibition of LFA-1. The results showed the role of neutrophils in Alzheimer's disease-like cognitive decline and pathology through LFA-1 integrin [94, 96]. Although NETs have been discovered in the AD brain, articles have failed to describe the relationship between NETs and AD [94, 96]. A*β*, as a DAMP, has been proven to be recognized by the complement system (CS) [94, 97]. Compliments such as C5a, CR1, and C1q may be related to NETs and AD [98]. Neutrophils may cause extrusion of NETs by recognizing A*β*. With the progression of inflammation and the accumulation of NETs, NETs trigger CS activation, especially C5a, via properdin binding and alternative pathways, eventually resulting in neurodegeneration [99, 100]. A study showed that there is a noticeable decline in the features of neuropathology and inflammation in an AD mouse model lacking C1q. Increases in C1q deposition suppress the activity of DNase, leading to the accumulation of NETs. After inhibition of C1q, the complement cascade reaction stops, and no NETs appear [101, 102]. Furthermore, the constituents of NETs damage neural cells within the brain parenchyma because they proteolytically activate inflammasome pathways and mitochondrial apoptosis and destroy extracellular matrix proteins [9]. The research on NETs is insufficient and needs further experimental elaboration.

### 4.5. NETs in SCI

SCI has many similarities with TBI. Typically, SCI is induced due to external physical impacts, called the primary injury. Violence causes an increase in interstitial pressure and haemorrhage, which press on the surrounding vessels and subsequently induce local ischaemia [103, 104]. The primary injury induces secondary injury, which further causes mechanical and chemical damage to spinal tissues, resulting in neuronal excitotoxicity due to the increasing level of reactive oxygen and high concentrations of calcium accumulated in cells [104]. Elevated levels of cytosolic Ca^2+^ enhance the activity of complex 1 and increase the production of ATP and ROS [104]. Early signs of TBI pathogenesis are tissue destruction, edema, and hypoxia, through which the earliest soluble mediators, ROS, are produced [67]. ROS are critical mediators in the formation of NETs. Therefore, we assume that SCI and TBI may share the same mechanism to induce NETosis. Apart from that, via the mitochondrial calcium uniporter, Ca^2+^ crossovers into the mitochondria. High levels of cytosolic Ca^2+^ induce increases in mPTPs and enhance membrane permeabilization [105]. Interestingly, mPTP is involved in NET formation and oxidative burst through activation of NADPH oxidase or other nonoxidative pathways [15]. Therefore, we reasonably speculate that NETs occur in the process of SCI. Sterile inflammation caused by spinal cord damage is characterized by infiltration of neutrophils in the spinal cord [106, 107]. As the main component in NETs, MPO is regarded as the primary cause of secondary injury due to its ability to generate HOCl [108, 109]. This cytotoxic substance strengthens apoptosis and necrosis, promotes the inflammatory response, and causes myelin degradation, thereby resulting in increased lesion size and further deteriorating neurological function [108, 109]. MPO exacerbates secondary injury and impairs functional recovery by enhancing neutrophil infiltration after SCI [110]. As an essential component of spinal cord edema, spinal cord astrocyte swelling is related to therapeutic resistance and poor functional recovery after SCI. Sun et al. [111, 112] revealed that an oxygen-glucose deprivation/reoxygenation (OGD/R) model improved the expression of HMGB1 and AQP4 and spinal cord astrocytic swelling, while HMGB1 inhibition by either ethyl pyruvate or HMGB1 shRNA led to a decrease in AQP4 expression and rat spinal cord astrocytic swelling after oxygen-glucose deprivation. HMGB1 upregulates AQP4 expression and promotes cell swelling in cultured spinal cord astrocytes after OGD/R, which is mediated through HMGB1/TLR4/MyD88/NF-*κ*B signalling and in an IL-6-dependent manner [111]. It has been reported that activation impacts are restrained by NF-*κ*B inhibition via the utilization of BAY 11-7082 or TLR4 inhibition via the utilization of C34 or CLI-095 [111, 112]. HMGB1 induces NETosis via TLR4 and exacerbates damage after cerebral ischaemia and cerebral thrombosis [85], and TLR4 is also an important signal for activating NETosis [112]. Therefore, we speculate that NET formation may mediate the process of injury in SCI, although evidence is still insufficient. However, some publications refute the damaging effect of neutrophils in SCI and hold the idea that the inflammatory function of neutrophils could reversely offer valuable outcomes for tissue repair in SCI [113]. There are still many unclear areas about neutrophil function in SCI. Therefore, further information about their cellular and molecular mechanisms of action is required to provide new insights in the field of SCI.

## 5. Therapy Methods of NETosis

Generally, NETosis has a greater impact on damage to CNS injuries, so preventing the progression of NETosis is vital to cure CNS injuries. Inhibition of NADPH oxidase, DNase, and PAD4 seems feasible to block NET formation, and we have provided details about potential drugs of NETosis in the table (Table 1). Apocynin (a naturally existing methoxy-substituted catechol) and VAS2870 (a triazolopyrimidine), which have been proven to be effective in primary human neutrophils, inhibit NADPH oxidase 2 (NOX2) and prevent the occurrence of NETosis induced by PMA and A23187 [15]. Inhibition of NADPH oxidase is rarely used in the clinic, and ROS produced by NADPH oxidase induces not only NETosis but also other harmful reactions. Therefore, we need to find a more specific medicine for application in the clinic. Recombinant DNase1 has been successfully implemented in pathological mouse models related to NETosis, such as SLE [114]. A formulation of DNase1 (Pulmozyme®) has been approved for cystic fibrosis treatment in NZB/WF1 hybrid mice [115]. Although the function of extracellular DNA extruded from neutrophils in CNS injury remains unknown, the application of DNase in autoimmune disease and mouse infection models has proven to be effective, yet further studies still need to explore the relationship between DNase and CNS injury. PAD4 prompts decondensation by catalysing citrullination of histones [32], and the binding of calcium and PAD4 enhances the bioactive conformation, causing the activity of PAD4 to be increased by 10,000 times [116]. F-amidine leads to irreversible inhibition of PAD4 by altering the active site of cysteine (Cys645), which is essential for catalysis [117, 118]. Cl-amidine is also a form of irreversible inhibitor that functions in the same manner as F-amidine [117, 119]. Both Cl-amidine and F-amidine have been shown to inhibit NETosis in mice [117, 118]. Moreover, the PAD4 inhibitory potency of Cl-amidine over F-amidine has also been proven in in vitro and in vivo experiments [118]. These compounds are extensively used in experiments to inhibit NET formation and are greatly effective. GSK484, a benzimidazole derivative, also reversibly inhibits PAD4, which tightly and reversibly attaches to the low-calcium (2 mM Ca) form of PAD4 and competes with the substrate. GSK484 is able to diminish histone citrullination and NET formation in mice treated with ionomycin [116, 118]. In addition, mPTP probably participates in NET formation, which is substantially decreased after using MeVal4CsA, bongkrekic acid (BKA), cyclosporine A (CsA), and sanglifehrin A (SfA) [15]. As an immunosuppressant, CsA inhibits mPTP opening by blocking the peptidyl-prolyl cis-trans isomerase (PPIase) activity of mitochondrial cyclophilin CypD, which is a critical regulatory component of the mPTP [15]. However, CsA inactivates the essential Ca^2+^-dependent protein phosphatase calcineurin, accordingly interrupting various signalling pathways [120–123]. MeVal4CsA and sanglifehrin A (SfA) have the same effect as CsA without the ability to inhibit calcineurin [120–123]. BKA is a form of adenine nucleotide translocase (ANT) inhibitor that inactivates translocase by freezing adenine nucleotide ANT in the matrix-oriented conformation, thereby suppressing the opening of mPTP [124]. All of these drugs have been proven to be effective in inhibiting NET formation in primary human neutrophils and CGD neutrophils [15]. Drugs related to NETosis inhibition are still rarely used in the clinic, so further studies are needed enable their use for the treatment of CNS injury.

## 6. Conclusion

In summary, NETosis is closely related to inflammatory reactions, and the function of damage outweighs the capability of repair in CNS injuries. The formation of NETs is boosted by diverse physiological stimuli, including lipopolysaccharides, protozoa, viruses, fungi, and bacteria. In addition, NETs are triggered by microcrystals, cholesterol, antibodies, specific cytokines (e.g., TNF-*α* and IL-8), and pharmacological stimuli containing calcium, potassium ionophores, and PMA. PAD4 is used to citrullinate histones, which will cause chromatin decondensation and NET release. NETs are the main components in the process of NETosis and have a negative effect on the CNS. We found that GSDMD and caspase-8 may be central points in NETosis, pyroptosis, necroptosis, and apoptosis. This will contribute to understanding the reciprocal relationships in all kinds of cell death in various diseases. In addition, we discuss the mechanism and existing evidence of NETosis in TBI, CNS infection, cerebral ischaemia, Alzheimer's disease, and SCI. TLR4, HMGB1, MPO, and NE have critical roles in these diseases. Finally, we expound on probable effective drugs to inhibit NETosis, in order to cure or alleviate CNS injury according to the NETosis mechanism, but most drugs are mainly used in experimental form, and few have progressed to the clinic.

Although NETosis is divided into suicidal NETosis and vital NETosis, the form that occurs in CNS injury remains unknown. DNA is vital to the viability of cells, and those in suicidal NETosis die because of the loss of DNA, while cells in vital NETosis remain alive after the release of NETs. We speculate that DNA in NETs may come from mitochondrial DNA; however, some cells will survive without the presence of nuclear DNA. Therefore, we wanted to know the exact mechanism of vital NETosis and whether the effects and events that occur between vital NETosis and suicidal NETosis are different. Moreover, we always discuss the effect of NETosis based on the function of NETs, but whether NETosis directly activates other cell death pathways or rather produces associate effective proteins or substances that contribute to the damage functions is still unclear. In this review, we discuss the function of NETosis in CNS injury and found much evidence about the occurrence of NETosis in CNS injury. However, what triggers NETosis is still unclear. TLR4 may be a recognized receptor, but whether other receptors recognize injury signals remains unknown. In addition, we focused on the damage of NETs to neurons but did not know whether NETosis influences other CNS cells. For example, if NETs can contribute to recovery of the spinal cord after SCI, did NETs activate Schwann cells or other cells? Or do NETs aggravate damage of other cells? Moreover, even though various biological events triggering the release of NETs have been excessively explored at present, their regulatory mechanisms are still unclear. In addition, related studies in vivo are rare. With regard to drugs, most inhibition of NETosis is mainly applied in experiments but rarely used in the clinic, and exploring the concrete mechanism of NETosis is fundamental on clinical trials. Therefore, further investigations are required to shed sufficient light on the underlying processes. We believe that studies concerning targets of NETosis will contribute to the therapy of a variety of diseases in the future.

## Figures and Tables

**Table 1:** Potential drugs targeted to NETosis.

Drugs	Target	Model	Potential mechanism	Reference
Apocynin	NOX2	Primary human neutrophils	Decrease production of ROS by inhibiting NOX2	[15]
VAS2870	NOX2	Primary human neutrophils	Decrease production of ROS by inhibiting NOX2	[15]
DNase1	DNA	NZB/W F1 hybrid mice	Endonuclease to digest extracellular DNA and degrade NETs	[114]
Cl-Amidine	Cys645	Mice	Irreversible inhibition of PAD4	[117, 118]
F-Amidine	Cys645	Mice	Irreversible inhibition of PAD4	[117, 118]
GSK484	Low-calcium (2 mM Ca) form of PAD4	Mice	Reversible inhibition of PAD4	[116, 118]
CsA	PPIase	Primary human neutrophils and CGD neutrophils	Inhibit mPTP open by blocking the PPIase activity of mitochondrial cyclophilin CypD	[15]
SfA	PPIase	Primary human neutrophils and CGD neutrophils	Inhibit mPTP open by blocking the PPIase activity of mitochondrial cyclophilin CypD	[15]
MeVal4CsA	PPIase	Primary human neutrophils and CGD neutrophils	Inhibit mPTP open by blocking PPIase activity of mitochondrial cyclophilin CypD	[15]
BKA	ANT	Primary human neutrophils and CGD neutrophils	Inhibit mPTP open by freezing adenine nucleotide ANT in the matrix-oriented conformation	[124]

## Data Availability

The datasets used and analysed during the current study are available from the corresponding authors on reasonable request.

## Abbreviations

AD: Alzheimer's disease
BBB: Blood-brain barrier
CBF: Cerebral blood flow
CCI: Controlled cortical impact
CGD: Chronic granulomatous disease
CNS: Central nervous system
CS: Complement system
CSF: Cerebrospinal fluid
CXCL: Chemokine (C-X-C motif) ligand
DAI: Diffuse axonal injury
DAMP: Damage-associated molecular pattern
DPI: Diphenylene iodonium
fMLP: N-Formyl-L-methionyl-L-leucyl-L-phenylalanine
GM-CSF: Granulocyte-macrophage colony-stimulating factor
GO: Glucose oxidase
Grx1: Glutaredoxin 1
GSDMD: Gasdermin D protein
HMGB1: High-mobility group box-1
IFN: Interferon
LFA-1: Lymphocyte function-associated antigen 1
MAPK: Mitogen-activated protein kinase
MEK: Mitogen-activated protein kinase
MMP: Metalloproteinase
MPO: Myeloperoxidase
mPTP: Mitochondrial permeability transition pore
NE: Neutrophilic elastase
NETs: Neutrophil extracellular traps
NLRP: NOD-like receptor family pyrin domain containing 3
NOX2: NADPH oxidase 2
OGD/R: Oxygen-glucose deprivation/reoxygenation
PAD4: Peptidylarginine deiminase 4
PAF: Platelet-activating factor
PI3K: Phosphoinositide 3-kinase
PKC: Protein kinase C
PMA: Phorbol 12-myristate 13-acetate
PMN: Polymorphonuclear neutrophil
PRR: Pattern recognition receptors
PSH: Paroxysmal sympathetic hyperactivity
PVN: Paraventricular nucleus
RIPK: Receptor-interacting serine-threonine kinase
ROS: Reactive oxygen species
SCI: Spinal cord injury
SLE: Systemic lupus erythematosus
TBI: Traumatic brain injury
TLR: Toll-like receptors
VWF: von Willebrand factor.
